# Comparing the Physiological Responses to the 6-Minute Walk Test, Timed Up and Go Test, and Treadmill Cardiopulmonary Exercise Test

**DOI:** 10.1155/2024/1317817

**Published:** 2024-09-30

**Authors:** Eduardo S. Alves, R. Nicole Bellet, Pramod Sharma, Bryce N. Balmain, Craig Aitken, Thomas Doering, Leilani Orola, Anita Green, Tatiana Paim, Fergus O'Connor, Norman R. Morris

**Affiliations:** ^1^ University Centre for Rural Health (UCRH) School of Health Sciences University of Sydney, Lismore, New South Wales, Australia; ^2^ Programa de Pós-Graduação em Ciências da Saúde Universidade Estadual de Santa Cruz, Ilhéus, Bahia, Brazil; ^3^ School of Allied Health Sciences and Social Work Griffith University, Southport, Queensland, Australia; ^4^ Physiotherapy Department The Prince Charles Hospital, Chermside, Brisbane, Queensland, Australia; ^5^ Institute for Exercise and Environmental Medicine Texas Health Presbyterian Hospital Dallas and Department of Internal Medicine University of Texas Southwestern Medical Center, Dallas, Texas, USA; ^6^ Allied Health Research Collaborative The Prince Charles Hospital, Chermside, Brisbane, Queensland, Australia; ^7^ School of Health Medical and Applied Sciences Central Queensland University, Rockhampton, Queensland, Australia; ^8^ Cardiac Investigation Unit The Prince Charles Hospital, Chermside, Brisbane, Queensland, Australia; ^9^ School of Human Movement and Nutrition Sciences University of Queensland, Brisbane, Queensland, Australia

**Keywords:** 6-min walk test, cardiopulmonary exercise test, timed up and go test, treadmill

## Abstract

**Purpose:** To compare physiological responses during a treadmill cardiopulmonary exercise test (CPX), 6-minute walk test (6MWT), and timed up and go test (TUGT) in individuals referred for unexplained breathlessness and symptom limited treadmill exercise testing.

**Methods:** Heart rate (HR), oxygen consumption (V̇O_2_), carbon dioxide production (V̇CO_2_), respiratory exchange ratio (RER), minute ventilation (V̇_E_), systolic blood pressure (SBP), and rating of perceived exertion (RPE) were recorded throughout each test.

**Results:** Each test demonstrated a significant increase (*p* < 0.01) in the cardiopulmonary (V̇O_2_, V̇CO_2_ and V̇_E_, RPE, SBP, and HR) and perceptual (RPE) responses from rest to end exercise. The increase in cardiopulmonary and perceptual responses was greatest for the CPX with significantly smaller responses demonstrated during the 6MWT (*p* < 0.01) and even smaller responses for the TUGT (*p* < 0.01 vs CPX and 6MWT).

**Conclusion:** Not surprisingly, the treadmill CPX results is the greatest physiological response in our group. Despite being of short duration, the TUGT results in an increased physiological response.

## 1. Introduction

Cardiopulmonary exercise testing (CPX) is a gold standard, noninvasive method used to assess cardiopulmonary fitness and exercise limitations [1] in both health and disease. The CPX is an incremental ramp test designed to provide a comprehensive assessment of functional capacity and highlight limiting mechanism(s) during exercise, which can be used to evaluate cardiovascular risk and prognosis in general and clinical populations [1, 2]. In addition, CPX is a maximal exercise test with concurrent gas exchange assessment which gives accurate and reproducible information about how ventilation, gas exchange, cardiovascular and skeletal muscle function interact and allows for the detection of abnormalities [2, 3]. The examination of both submaximal and peak exercise responses is possible with the use of this dynamic, relatively noninvasive physiologic test, which also offers pertinent data for therapeutic decision-making [3].

Unfortunately, performance of the CPX is impractical in many settings due to the requirement for specialised equipment, trained staff, and medical supervision, especially if the only purpose of the test is to allow admission into cardiopulmonary rehabilitation [1–3]. Approximately two thirds of cardiopulmonary rehabilitation clinics in the United States do not report measuring baseline CPX, although this method is the gold standard for exercise programming in cardiopulmonary rehabilitation [4].

Consequently, several alternative tests to the CPX have been used to assess exercise capacity, provide parameters to estimate training requirements, and as an outcome measurement for the evaluation of research and physical exercise programs [5], although providing a less comprehensive assessment of limiting mechanism(s) for exercise. One such test is the 6-minute walk test (6MWT), which is widely employed across pulmonary and cardiac rehabilitation programmes [6, 7]. Compared to the CPX, the 6MWT benefits in that it is low cost, is easy to implement, and involves an activity commonly performed by patients, that is, walking on flat ground [6].

The similarity of the physiological responses achieved during the 6MWT compared to those achieved during a CPX varies in different populations. In some cardiopulmonary populations such as heart failure, [8] pulmonary hypertension [9], and interstitial lung disease [10], the physiological responses measured during the CPX and the 6MWT appear similar. However, in middle-older aged healthy adults, the 6MWT produced approximately 80% of the oxygen consumption (V̇O_2_) generated during a CPX [11]. Possible reasons for these different responses could be that in populations with severe disease and underlying cardiac/ventilatory limitations, the 6MWT evokes a maximal exercise response, while only evoking a submaximal response in populations with mild or no functional restrictions [8, 12]. Further, when comparing the 6MWT to cycle- rather than treadmill ergometer, the CPX responses may differ dependent upon muscle recruitment during the tests [13]. In this context, studies comparing treadmill CPX and 6MWT physiological responses in cardiac populations are important to identify any commonalities and differences between the two tests.

The 6MWT distance (6MWD) has been found to have a moderate to strong association with relative peak V̇O_2_ and has been shown to be useful for predicting health outcomes in heart failure [14–16], in older people [17] and people with pulmonary disease [18], although such results may differ in other populations. For example, 6MWD is a strong predictor of death and poor prognosis in heart failure, with each 50 m decrease in 6MWD associated with a 20% risk of death or hospital readmission [14–16]. A 6MWD between 290 and 338 m has been associated with an increased risk of all-cause mortality [17], and in people with pulmonary hypertension, a 6MWD greater than 400 m is associated with a reduced risk of a pulmonary hypertension related hospitalisation or death at 6 months [19].

However, some individuals have physiological or functional limitations that inhibit their ability to perform a 6MWT or a CPX [20, 21]. For these individuals, a timed up and go test (TUGT) has been proposed as an alternative test in clinical settings [22]. The TUGT was initially created to measure balance, walking speed, and functional ability [23]. As balance, age, and strength play a role in determining walk distance, [24] the TUGT has been considered a potential alternative outcome measure to the 6MWT, particularly in the home setting [22]. In addition, Ascencio et al. consider that the TUGT has the ability to explore the interactions between cardiopulmonary, nervous, and musculoskeletal systems involved in it [25]. For example, one retrospective cohort study found that slower TUGT time was associated with increased mortality risk and cardiovascular disease in older adults [26]. A TUGT time higher than 10 s resulted in a 2.9-fold increase in cardiovascular mortality in Korean women (66 years) [27], and TUGT time was positively associated with higher risk of all-cause mortality in a population of older people (60+) in Peru [25]. Moreover, Bellet et al. found similar relative changes in the TUGT and the 6MWT in 154 patients following a cardiac rehabilitation program and suggested the TUGT may provide a related or supplementary measure of functional capacity in cardiac rehabilitation [22].

With its typically short duration, the physiological response to the TUGT is not typically documented, despite some literature suggesting the TUGT may have importance in measuring mortality, risk of disease, and exercise capacity. To our knowledge, no studies have described the physiological responses of TUGT or compared these to a CPX or 6MWT.

Therefore, the purpose of the current study was to compare the cardiopulmonary physiological responses (cardiopulmonary and perceptual) of three tests used to measure clinical exercise capacity, that is, the CPX, 6MWT and TUGT, in a group of individuals referred for cardiac investigations and compare the relationship between these tests. It was hypothesised that 6MWD and TUGT duration would be correlated with end exercise V̇O_2_ measured during the CPX. Further, the TUGT would elicit an increase in cardiopulmonary responses compared to rest but with a substantially lower end exercise response compared to CPX and 6MWT.

## 2. Methods

### 2.1. Study Design and Setting

This is a descriptive study within-subjects design with comparisons between three different tests (treadmill CPX, 6MWT, and TUGT). This study was conducted at the Cardiac Sciences Unit at The Prince Charles Hospital (TPCH) where temperature (22 ± 1°C) and humidity (30%–40%) were controlled and stable. This study was approved (38437 HREC/17/QPCH/419) by the TPCH Human Research Ethics Committee.

### 2.2. Participants

A statistical power analysis was performed using previously published data [10] using the jpower module (v.0.1.2) in Jamovi (v.2.5.0), indicating that a minimum of 13 participants would be required to detect a 0.3 L.min^−1^ difference (1 metabolic equivalent (MET) for group) in end-exercise VO_2_ between testing modalities with ≥ 84% power (*α* = 0.05).

Nineteen participants referred to cardiac investigations, who undertook a treadmill CPX, completed this study. Included participants were aged over 18 years; able to walk on a treadmill without limitation; and provided written informed consent. Participants were excluded if they were deemed unsuitable for further walk tests by the medical officer supervising CPX; unable to perform the CPX; or unable to perform the TUGT or 6WMT.

### 2.3. Procedures

Participant characteristics (age, gender, medical history, prescribed medications, height, body mass, resting heart rate (HR), and resting blood pressure) were collected immediately prior to testing. Following the CPX, participants were asked to return to the hospital within a week to perform TUGT and 6MWT. Participants were asked to wear a portable breath by breath metabolic system (Metamax 3B, Cortex, Leipzig, Germany) using a flexible Velcro harness, easy to fit on the back or in front, allowing free movement with no discomfort while undertaking the CPX, TUGT, and 6MWT to collect indirect calorimetry data. The portable metabolic system was calibrated for expired volume and gases (oxygen and carbon dioxide) immediately prior to each test. The portable metabolic system is both reliable and valid for the measurement of pulmonary gas exchange during exercise [28]. In a recent comparison of 15 metabolic systems, the Cortex Metamax 3B had a < 1.7% measurement error for V̇O_2_ compared to metabolic simulation values [28].

Throughout these three tests, breath by breath analysis of gas exchange, HR and arterial oxygen saturation (S_p_O_2_) (RAD-5v Masimo Corp, Irvine, CA, United States) were monitored continuously. V̇O_2_, carbon dioxide production (V̇CO_2_), respiratory exchange ratio (RER), and minute ventilation (V̇_E_) were recorded at rest, during and at end exercise (Metamax-Analysis 3B, Cortex, Leipzig, Germany). For the CPX and the 6MWT, metabolic variables were averaged over 30 s periods. Systolic blood pressure (SBP) and diastolic blood pressure (DBP) were measured by a sphygmomanometer (Welch Allyn 767 Series mobile aneroid sphygmomanometer, Skaneateles Falls, NY, United States) and stethoscope at rest and end exercise. Rating of perceived exertion (RPE), using the 6–20 original Perceived Exertion Borg Scale [29], was measured at rest and end exercise.

### 2.4. CPX

The CPX was performed on a treadmill (T2100-ST2, GE Healthcare Wauwatosa, WI, United States) using electrocardiographic monitoring (GE Healthcare CASE V6.73 Wauwatosa, WI, United States) and followed standard guidelines [30] for either the standard Bruce protocol [31] or the modified Bruce protocol, [32] depending upon medical referral received. End exercise V̇O_2_, V̇CO_2_, and V̇_E_ were taken from the averaged data during the last 30 s of the test.

### 2.5. 6MWT

The 6MWT was conducted following standard guidelines [5, 6] along a flat, 30-m, indoor track. Assessors used a script with standardised encouragement and recorded the 6MWD; rests required; and any adverse symptoms experienced by participants during each test. Each participant completed two 6MWTs on the same day, separated by at least 10 min of seated rest until HR, BP, and RPE returned to baseline. Data from the best test (highest 6MWD) were used for analysis. End exercise V̇O_2_, V̇CO_2_, and V̇_E_ were considered the averaged data during the last 30 s of the test.

### 2.6. TUGT

The TUGT was conducted using a chair with arms and a seat height of 46 cm placed on a flat, indoor walking track with cones marking the 3-m turning point [33]. Participants were instructed, “On the word ‘go', get up and walk as quickly and as safely as possible to cross the line marked on the path, turn around, walk back, and sit down.” The activity was timed from when the participant's back left the chair to the return of the participant's back to the chair. Participants performed one untimed practice TUGT followed by two timed TUGTs. At least 4 min of seated rest occurred between each TUGT. The TUGTs were conducted before the 6MWT to limit the variable effects of fatigue on participants and to minimise the disruption to physiological measurements. Metabolic data was collected continuously during and for at least 2 min immediately following the TUGT. Due to the short duration of the TUGT, cardiopulmonary data was averaged over 10 s intervals. The highest 10 s average V̇O_2_, HR, V̇CO_2_, and V̇_E_ recorded immediately (within 60 s) after the TUGT completion was taken as the end exercise value. The lowest duration obtained was reported from the two timed tests.

### 2.7. Statistical Analyses

Linear mixed effects models were utilised to determine the influence of testing modality on all outcome variables of interest. Testing modality (CPX [reference test], 6MWT, and TUGT) and timepoint (baseline resting and end-exercise) were included as fixed effects, and a random intercept was included to account for repeated observations within each participant.

The normality of the distribution of residuals was assessed via visual inspection of histograms and QQ plots as well as via formal tests of normality (Shapiro–Wilk and Kolmogorov–Smirnov tests). Due to non-normality of residuals, data was log transformed. Linear mixed effects models with the Bonferroni post hoc comparisons, utilising the GAMLj module in Jamovi (v.2.5.0), were undertaken on the logarithm of all outcome variables of interest.

Given the duration of the CPX varied from participant to participant, the data was divided into quartiles and compared with quartile data from 6MWT. In this way, each quartile or interval includes 25% of the total data, arranged from smallest (baseline rest) to largest (Q100). We excluded the TUGT from this analysis due to its short duration. Comparisons between CPX and 6MWT variables at the same quartile were performed using similar methods to those described above whereby testing modality (CPX [reference test] and 6MWT) and timepoint (Q1, Q2, Q3, and Q4) were included as fixed effects, and a random intercept was included to account for repeated observations within each participant. In addition, linear mixed effects models were used to compare differences based on data collection quartiles (baseline rest, Q25, Q50, Q75, and Q100) within the same test where timepoints (baseline rest, Q1, Q2, Q3, Q4) were included as fixed effects, and a random intercept was included to account for repeated observations within each participant. All quartile data was log transformed prior to statistical analysis. Finally, the Spearman correlations were used to assess the associations between V̇O_2_ at the completion of the CPX, 6MWT, and TUGT duration. Values are presented as median (interquartile range), minimum, and maximal. The significance level adopted was *p* < 0.05.

## 3. Results

Nineteen participants (11 males) consented to participate and completed all experimental conditions.  Table 1 shows the general characteristics of these participants and the end exercise results and a broad range of age, BMI, and response to exercise.

Table 2 shows the cardiopulmonary pretest (rest), post-test (end), and delta values during the CPX, 6MWT, and TUGT. Aside from SpO_2_, testing modality significantly influenced all outcome variables of interest, such that RPE, SBP, DBP, HR, relative V̇O_2_, absolute V̇O_2_, V̇CO_2_, and V̇_E_ were significantly greater for CPX compared with TUGT and the 6MWT (all *p* ≤ 0.013). Similarly, all outcome variables of interest aside from SpO_2_ (*p* = 0.181) were significantly influenced from baseline resting to end exercise (all *p* ≤ 0.001). Moreover, all variables aside from DBP were greater in the CPX compared to the 6MWT and TUGT (all *p* ≤ 0.001).

**Figure 1:**
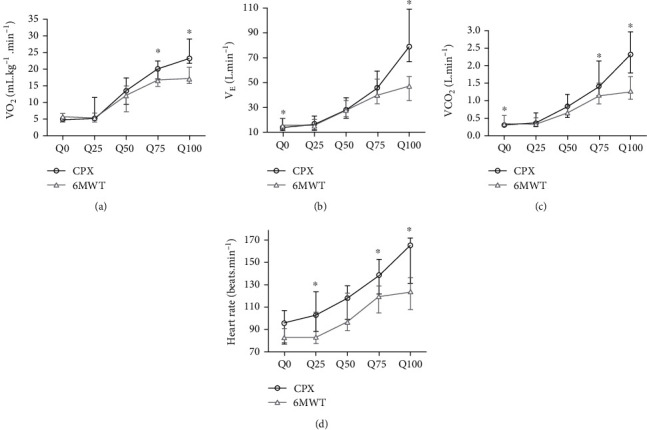
Values of (a) V̇O_2_, (b) V̇_E_, (c) V̇CO_2_, and (d) HR at baseline rest (Q0), quartile 25 (Q25), quartile 50 (Q50), quartile 75 (Q75), and quartile 100 (100) during cardiopulmonary exercise testing (CPX) and 6-minute walk test (6MWT) in the participants (*n* = 19) of the study. Results are expressed in median and interquartile range. The significance level adopted was *p* < 0.05. Wilcoxon test was used to compare values of relative V̇O_2_, V̇CO_2_, V̇_E_, and HR between CPX and 6MWT at the same quartile. ∗Different from CPX, *p* < 0.05. V̇O_2_ = oxygen consumption; V̇_E_ = minute ventilation; V̇CO_2_ = carbon dioxide production; HR = heart rate.

Figure 1 shows the quartile values of V̇O_2_, V̇CO_2,_ V̇_E_, and HR during CPX and 6MWT. V̇O_2_ was significantly greater at Q75 and Q100 (both *p* < 0.005) during CPX compared to the 6MWT. Similarly, V̇_E_ was significantly higher at Q100 (*p* < 0.005) during CPX compared to the 6MWT. V̇CO_2_ was significantly higher at Q50, Q75 and Q100 (all *p* < 0.005) during CPX than compared to the 6MWT. Finally, HR was significantly higher at Q25, Q75, and Q100 (all *p* < 0.005) during CPX 6MWT.

Regarding comparisons of data collection quartiles (baseline rest, Q25, Q50, Q75, and Q100) within CPX; V̇O_2_, V̇_E_, V̇CO_2_, and HR values were significantly influenced by quartile. Post hoc comparisons showed that V̇O_2_, V̇_E_, V̇CO_2_, and HR values at Q100 were higher compared to Q75, Q50, Q25, and baseline rest (all *p* < 0.001). V̇O_2_, V̇_E_, V̇CO_2_, and HR values at Q75 were higher than those observed at Q50, Q25, and baseline rest (all *p* < 0.042). In addition, V̇O_2_, V̇_E_, and V̇CO_2_ values at Q50 were higher than those observed at Q25 and baseline rest (both *p* < 0.001). V̇O_2_ and V̇CO_2_ values at Q25 were greater than those observed at baseline rest (*p* ≤ 0.023). In contrast, there was no difference between HR values between Q50 and Q25 (*p* = 0.712), or V̇_E_, and HR values at Q25 and baseline rest (all *p* ≥ 0.06).

Regarding comparisons of data collection quartiles (baseline rest, Q25, Q50, Q75, and Q100) within the 6MWT; V̇O_2_, V̇_E_, V̇CO_2_, and HR values were significantly influenced by quartile. Post hoc comparisons showed that V̇O_2_, V̇_E_, V̇CO_2_, and HR values at Q75 were higher than those observed at Q50, Q25, and baseline rest (all *p* ≤ 0.024). V̇O_2_, V̇_E_, V̇CO_2_, and HR values at Q50 were higher than those observed at Q25 and baseline rest (all *p* ≤ 0.004). In addition, V̇O_2_ values at Q25 were greater than those observed at baseline rest (*p* = 0.011). In contrast, there was no difference between V̇O_2_, V̇_E_, V̇CO_2_, and HR values at Q100 and Q75 (all *p* > 0.99), or V̇_E_, V̇CO_2_, and HR values at Q25 and baseline rest (*p* > 0.98).

**Figure 2:**
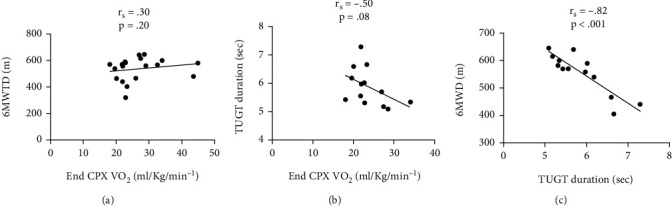
Spearman correlations values (*r*_*s*_) between (a) 6MWD and end CPX relative V̇O_2_, (b) TUGT durations and end CPX relative V̇O_2_, and (c) 6MWD and TUGT duration. The significance level adopted was *p* < 0.05. V̇O_2_ = oxygen consumption; CPX = cardiopulmonary exercise test; 6MWD = 6-minute walk test distance; TUGT = timed up and go test.

While there was no significant relationship between CPX end V̇O_2_ and 6MWD and between CPX end V̇O_2_ and TUGT, there was a strong negative correlation (*r* = −0.82; *p* < 0.001) between 6MWD and TUGT duration (Figure 2).

## 4. Discussion

Our study is the first to compare three tests used to measure clinical exercise capacity, that is, the CPX, 6MWT, and TUGT, using breath-by-breath analysis of gas exchange in a group of individuals referred for cardiac investigations for unexplained breathlessness, and to investigate the relationship between these tests.

We found, as hypothesised, that subjects undertaking both the TUGT and the 6MWT had significantly increased cardiopulmonary responses compared to rest. Further, end exercise responses were significantly lower during 6MWT and significantly lower again during TUGT when compared to CPX. However, in our participants (Table 1), TUGT elicited 52%, 30%, and 72%, respectively, of the median peak HR (b/min), V̇O_2_ (ml/kg/min^−1^), and RER achieved during CPX. Similarly, 6MWT elicited 73%, 75%, and 85%, respectively, of the median peak HR, V̇O_2_ (ml/kg/min^−1^), and RER achieved during CPX. While we found a strong negative correlation between 6MWD and TUGT duration (r_s_ = −0.82; *p* < 0.001), we found no correlation between CPX end V̇O_2_ and 6MWD or between CPX end V̇O_2_ and TUGT duration.

In heart failure, [8] pulmonary hypertension [9], and interstitial lung disease [10], the physiological responses measured during the CPX and the 6MWT have been shown to be comparable, while middle-older aged healthy adults undertaking a 6MWT attained only 80% of the V̇O_2_ generated during a CPX [11]. Our study corroborates these previous results for the 6MWT [11, 34]. It is possible that responses to the 6MWT, in subjects with established chronic conditions, may correspond more closely to the CPX. While the participants in our study had no identified underlying pathology, they appeared to have reduced exercise capacity when compared to published data from similarly aged individuals. By way of comparison, participants in the current study had lower end exercise V̇O_2_ values during the CPX when compared to healthy people [35, 36] (age 50–59 years, ~33.5 mL.kg^–1^.min^–1^) and similar values to people with cardiovascular disease (age 50–59 years, ~21 mL.kg^–1^.min^–1^) [37]. In addition, the 6MWD achieved (567 m) in the current study appears lower than Australian healthy subjects (age 55–75 years, 659 ± 62 m) [38], yet similar to healthy subjects (age 40–80 years, 571 ± 90) from seven countries [39]. Similarly, the TUGT duration of 5.6 s in our study is below the value reported for individuals aged 60^+^ years [40]. Therefore, it is likely that individuals referred for further cardiac investigation in our study have a below average exercise capacity.

Performance during standardised exercise tests (laboratory and field tests) and their corresponding physiological responses are of known value in the comprehensive evaluation of heart and respiratory disorders [1, 41]. In the present study, we found a significant increase in the physiological (except diastolic pressure) responses in all three tests from rest to end exercise (Table 2). Although changes in these variables during the CPX and 6MWT were expected, [41], this is the first study to show that TUGT leads to an increase in the metabolic parameters, RPE, and hemodynamic responses compared to rest. However, despite statistically significant changes, the magnitude of change during the TUGT in our sample was very small, for example, V̇O_2_ increased by ~1 MET, and RPE increased to a median of seven, suggesting this test elicits only a minor physiological challenge, likely due to the short duration of the test. The effect of both duration and intensity of exercise or a dose-response may also explain the findings of the current study. This likely reflects a “dose-response” phenomenon whereby the short duration of the TUGT could not elicit the magnitude of stimulus observed during the longer duration on CPX and 6MWT. In addition, the absolute intensity of exercise during CPX is clearly higher compared with 6MWT and TUGT. Combining the longer duration and greater intensity of exercise would suggest that the CPX provides a greater dose of exercise, when compared to the 6MWT and TUGT, resulting in consequently higher values towards the end of the exercise test for the RPE, metabolic, and cardiovascular variables measured in our study [42].

The acute physiological responses to exercise depend on the type, intensity, and duration of the exercise performed [41, 43]. Although all three testing modalities utilised in the current investigation are walking tests, the CPX is characterised by longer duration and higher external workload (higher intensity) than the 6MWT and TUGT, which appear likely to have resulted in higher physiological responses observed in the present study. In addition, the speed of the 6MWT and TUGT is participant-determined and led to a plateauing of the exercise intensity once a fast walk (approximately 5–6 km/h) was achieved. Notably, the disparate relative intensities of the testing modalities utilised are likely reflected within the perceptual responses of participants, whereby RPE was greater during the CPX, compared to the 6MWT and TUGT. This is an important finding and suggests that similar to findings in individuals with common chronic conditions [44], the association between perceived and actual exertion may not be blunted in individuals with unexplained breathlessness. However, this notion requires further investigation.

In spite of the fact that the 6MWT and TUGT are typically assumed to be submaximal tests, studies have found that in some clinical populations the 6MWT evokes similar physiological responses to those observed during CPX, suggesting peak oxygen uptake can be obtained during short duration tests (i.e., 6MWT) [8, 9, 12]. Given that the CPX and 6MWT are typically of different durations, we divided the CPX and 6MWT in quartiles to examine the kinetics of variables of interest in order to enable comparison at a fixed relative point in the test. In the present study, the physiological responses for CPX and 6MWT were similar during the first half of the tests (lower intensities). However, despite the low levels of exercise capacity in our study participants [35, 36], the 6MWT did not evoke physiological response comparable to that in the treadmill CPX. The 6MWT can be described in our sample as a moderate to high intensity exercise, which elicited a HR and V̇O_2_ of approximately 75%–80% of the peak measured on CPX. Also, other variables such as V̇_E_ and V̇CO_2_ had similar kinetics to HR and V̇O_2_ with a significantly higher value during the second half of the tests.

The end exercise V̇O_2_ in the CPX, 6MWD, and TUGT duration have been used as outcome measures during cardiopulmonary rehabilitation [14–16, 22, 26] and previous studies have investigated the association between these variables [22, 45–48]. While some studies found correlations between CPX peakV̇O_2_ and 6MWD [34, 47, 48], the present study found no correlation between these variables. Similar to our results, no correlation between 6MWD and CPX peak V̇O_2_ was observed in advanced heart failure [45] and in young healthy individuals [46]. We also found no correlation between CPX end V̇O_2_ and TUGT duration. However, we found a strong negative correlation between 6MWD and TUGT duration. Similarly, Bellet et al. [22] found a moderate negative correlation between 6MWD and TUGT duration in 61 community-based cardiac rehabilitation patients, consistent with our results. While the physiological response to the TUGT remains minimal, the test duration is strongly (negatively) correlated with the 6MWD. That being, as TUGT duration decreases (i.e., performance increases), 6MWD increases. Thus, the TUGT may provide a simple, alternative measure of the effectiveness of cardiopulmonary rehabilitation programs, particularly for those individuals unable to perform the 6 min of walking required for the 6MWT.

This study has some limitations that should be considered when interpreting the data. First, the three tests administered were not randomised, and we did not control confounding variables, which may introduce some bias in our results. However, this was a necessary limitation of the testing protocol whereby participants were recruited after referral to the Cardiac Investigation Unit at The Prince Charles Hospital. Also, for more specific observations, a larger number of subjects could be divided into different subgroups. Finally, the results found in the present study should be interpreted with caution and may be different in people with cardiovascular or respiratory diseases.

In conclusion, an increase in physiological responses was elicited immediately following TUGT, although this response was small compared to that following CPX and 6MWT in individuals referred for symptom limited treadmill due in the current study. Further, while the 6MWD and TUGT duration were not correlated to CPX end V̇O_2_ in our sample, it is possible that such correlations may be found in populations with much lower exercise capacity (e.g., people with chronic diseases). Our results suggest that the submaximal 6MWT and TUGT do have a place where access to CPX is not available to determine safe exercise prescription and for outcome measurement. Assessment of exercise capacity using the 6MWT and TUGT is enhanced by measurement of HR, SBP, DBP, RPE, and SpO2 and the use of portable breath-by-breath analysis of gas exchange if available. Finally, participants with shortest TUGT time may have better 6MWT capacity.

## Figures and Tables

**Table 1:** Participant characteristics and end exercise responses.

**Variables**	**Median (interquartile range)**	**Min and max**
Age (years)	51 (40, 68)	23–75
Height (cm)	172 (170, 178)	154–185
Body mass (kg)	82 (74, 105)	60–180
Body mass index (kg.m^−2^)	27.7 (25.9, 34.2)	20–52
SBP (mmHg)	130 (120, 140)	110–150
DBP (mmHg)	80 (80, 85)	70–110
End exercise: cardiopulmonary exercise test (CPX)		
V̇O_2_ (mL.kg^−1^.min^−1^)	23.2 (21.8, 29)	18–44.8
HR (beats.min^−1^)	169 (152, 180)	145–197
RER	1.07 (0.95, 1.11)	0.77–1.29
End exercise: 6-minute walk test (6MWT)		
6MWD (m)	567 (466, 590)	320–645
V̇O_2_ (mL.kg^−1^.min^−1^)	17.3 (16.1, 20.8)	9.52–35.8
HR (beats.min^−1^)	123 (108, 136)	76–145
RER	0.91 (0.84, 0.96)	0.69–1.04
End exercise: timed up and go test (TUGT)		
Duration (s)	5.6 (5.3, 6.3)	5–7.2
V̇O_2_ (mL.kg^−1^.min^−1^)	6.9 (5.6, 8.1)	4.51–11.5
HR (beats.min^−1^)	88 (77, 102)	50–105
RER	0.77 (0.68, 0.82)	0.52–0.94

*Note:* Results are expressed in median, Quartiles 1 and 3 (between brackets separated by a coma), minimum and maximum.

Abbreviations: 6MWD, 6-minute walk distance; 6MWT, 6-minute walk test; CPX, cardiopulmonary exercise testing; DBP, Diastolic blood pressure; HR, Heart rate; kg.m^−2^, kilogram/meters square; mmHg, millimetre of mercury; RER, respiratory exchange ratio; SBP, systolic blood pressure; TUGT, timed up and go test; V̇O_2_, oxygen consumption.

**Table 2:** Pretest (rest), post-test (end), and delta (Δ) values for metabolic, haemodynamic, and RPE from pre-exercise to end during the cardiopulmonary exercise, 6-minute walk, and timed up and go tests.

	**V̇O**_**2**_ ** (ml.kg** ^**−1**^ **.min** ^**−1**^ **)**	**V̇O**_**2**_ ** (L.min** ^**−1**^ **)**	**V̇CO**_**2**_ ** (L.min** ^**−1**^ **)**	**V̇**_**E**_ ** (L.min** ^**−1**^ **)**	**SpO**_**2**_ ** (%)**	**HR (beats.m** ^**−1**^ **)**	**RPE**	**SBP (mmHg)**	**DBP (mmHg)**
CPX									
Rest	4.8 (4.1, 5.2)	0.42 (0.34, 0.48)	0.29 (0.26, 0.33)	13 (11, 15)	99 (98, 99)	96 (77, 107)	6 (6, 7)	130 (120, 140)	75 (70, 80)
End	23.2 (21.8, 29.0)	2.11 (1.83, 2,56)	2.31 (1.70, 2.90)	78 (66, 109)	99 (97, 100)	166 (132, 172)	17 (15, 18)	180 (170, 200)	70 (65, 80)
Δ	19 (16,23)	1.59 (1.52, 3.03)	1.90 (1.50, 2.50)	60 (52, 91)	0 (0, 0)	65 (55, 80)	9 (8, 11)	55 (50, 68)	−7 (−15, −5)
6MWT									
Rest	5.8 (4.5, 6.6)	0.46 (0.35, 0.76)	0.33 (0.28, 0.57)	15 (12, 20)	98 (98, 99)	85 (78, 103)	6 (6, 7)	122 (118,130)	80 (78, 88)
End	16.3 (14.8, 18.8)	1.35 (1.12, 1.63)	1.18 (1.00, 1.50)	44 (35, 54)	98 (97, 99)	124 (109, 135)	10 (9, 12)	138 (128, 144)	80 (70, 80)
Δ	11 (9,14)	1.04 (0.79, 1.20)	0.80 (0.80, 1.20)	30 (22, 40)	0 (−1, 0)	33 (26, 43)	4 (3, 5)	11 (8, 19)	0 (−2, 1)
TUGT									
Rest	4.6 (3.9, 5.0)	0.35 (0.44, 0.45)	0.28 (0.25, 0.35)	13 (11, 14)	99 (98, 99)	82 (72, 96)	6 (6, 7)	124 (116, 130)	80 (76, 80)
End	6.9 (5.6, 8.1)	0.56 (0.50, 0.80)	0.42 (0.37, 0.50)	17 (15, 22)	98 (98, 99)	89 (77, 103)	7 (6, 9)	128 (118, 132)	80 (70, 80)
Δ	2.1 (1.7, 3.3)	0.23 (0.16, 0.29)	0.10 (0.10, 0.20)	4 (3, 6)	0 (0, 0)	4 (3, 7)	1 (0, 2)	2 (2, 6)	0 (0, 0)
*p* values									
Test	< 0.001	< 0.001	< 0.001	< 0.001	0.952	< 0.001	< 0.001	< 0.001	0.001
Time	< 0.001	< 0.001	< 0.001	< 0.001	0.181	< 0.001	< 0.001	< 0.001	< 0.001
Interaction	< 0.001	< 0.001	< 0.001	< 0.001	0.753	< 0.001	< 0.001	< 0.001	0.013

*Note:* Results are expressed in median (interquartile range).

Abbreviations: 6MWT, 6-minute walk test; CPX, cardiopulmonary exercise testing; DBP, diastolic blood pressure; HR, heart rate; mmHg, millimetres mercury; RPE; rating perceived exertion; SBP, systolic blood pressure; SpO_2_, oxygen saturation; TUGT, timed up and go test; V̇_E_, minute ventilation; V̇CO_2_, carbon dioxide production; V̇O_2_, oxygen consumption.

## Data Availability

The data that support the findings of this study are available from the last author, (N.R.M.), upon reasonable request.
